# Association of PKD1 sequence variants with pathophysiology of ADPKD in Indian patients

**DOI:** 10.1186/1755-8166-7-S1-P31

**Published:** 2014-01-21

**Authors:** Sonam Raj, RG Singh, Parimal Das

**Affiliations:** 1 Centre for Genetic Disorders, Faculty of Science, Banaras Hindu University, Varanasi, India; 2 Dept. of Nephrology, Institute of Medical Sciences, Banaras Hindu University, Varanasi, India

## Background

Autosomal dominant polycystic kidney disease (ADPKD) is a systemic conditions with cardinal symptom of bilateral multiple renal cysts of variable sizes. ADPKD is genetically heterogeneous, Mendelian disorder, shows late onset and contributes 8-10% cases of end stage renal disease (ESRD). Linkage analysis in ADPKD familial cases revealed approximately 85% cases of PKD1, rest cases of PKD2 and an unidentified locus.

## Materials & methods

In the present study occurrence of mutation, amino acid change and three frequently observed SNPs from 3’single copy region (exon 44-46) and in 5’ duplicated region (exon 2-11) of were screened in 47 patients (24 sporadic and 23 familial) and in control individuals to search the responsible and/or susceptible spots for ADPKD in Indian patients using direct DNA sequencing of specified exons and RFLP for SNP assay. PKD1 exons 44-46 encoding intracellular and exons 2-11 encoding ligand binding domains, Ig-like PKD domains in extracellular compartment with their suggested role in cell signaling and cell adhesion were chosen for this study.

## Results

Screening of exons 44-46 in 47 patients identified five exonic (I4045V, 4059V, A4092A, L4137L, P4210P) and two intronic (IVS44+15G>A, IVS+22delG) variants. Assessment of the three SNPs viz. I4045V, A4059V, and A4092A screened in a multiplex family with four affected (II-1, II-7, II-9, III-12) and 30 unaffected members along with 100 ages and sex matched controls showed higher occurrence (2X) in patients compared to controls indicating their role for disease susceptibility. Screening of exons 2-7 in 19 patients uncovered six exonic (A92A, c.445delC, T191N, G340G, N416N, A443T) and two intronic (IVS4+21delC, IVS6+12delC) variants while screening of exons 8-11 in 15 patients detected twelve exonic (Q554X, T558M, Q562R, A637V, P676L, c.1987delC+2016_2017insG, D910D, L811L, L845S, N854S, V873A, N890N) and one intronic (IVS9+14_27del14) variants. Two exonic changes (c.445delC, c.1987delC+2016_2017insG) generate 288 amino acids long truncated protein and a stretch of nine altered amino acids sequence in LDL-A domain respectively.

## Conclusions

The spectrum of changes detected in various affected individuals indicates that the clinical as well as allelic heterogeneity compounding the pathophysiology of ADPKD in both sporadic and familial cases. Screening of remaining region of PKD1 and other candidate genes is therefore planned for future for better understanding.

